# Antioxidant Effectiveness of Vegetable Powders on the Lipid and Protein Oxidative Stability of Cooked Turkey Meat Patties: Implications for Health

**DOI:** 10.3390/nu5041241

**Published:** 2013-04-17

**Authors:** Garry Duthie, Fiona Campbell, Charles Bestwick, Sylvia Stephen, Wendy Russell

**Affiliations:** Natural Products Group, Rowett Institute of Nutrition and Health, University of Aberdeen, AB21 9SB, Scotland, UK; E-Mails: fiona.campbell@abdn.ac.uk (F.C.); C.Bestwick@abdn.ac.uk (C.B.); Sylvia.Stephen@abdn.ac.uk (S.S.); W.Russell@abdn.ac.uk (W.R.)

**Keywords:** oxidative stability, turkey patties, vegetable powders, antioxidants

## Abstract

Lipid and protein oxidation decreases the shelf-life of foods and may result in formation of end-products potentially detrimental for health. Consumer pressure to decrease the use of synthetic phenolic antioxidants has encouraged identification of alternative compounds or extracts from natural sources. We have assessed whether inclusion of dried vegetable powders improves the oxidative stability of turkey meat patties. Such powders are not only potentially-rich sources of phenolic antioxidants, but also may impart additional health benefits, as inadequate vegetable consumption is a risk factor for heart disease and several cancers. In an accelerated oxidation system, six of eleven vegetable powders significantly (*p* < 0.05) improved oxidative stability of patties by 20%–30% (spinach < yellow pea < onion < red pepper < green pea < tomato). Improved lipid oxidative stability was strongly correlated with the decreased formation of protein carbonyls (*r* = 0.747, *p* < 0.01). However, improved lipid stability could not be ascribed to phenolic acids nor recognized antioxidants, such as α- and γ-tocopherol, despite their significant (*p* < 0.01) contribution to the total antioxidant capacity of the patties. Use of chemically complex vegetable powders offers an alternative to individual antioxidants for increasing shelf-life of animal-based food products and may also provide additional health benefits associated with increased vegetable intake.

## 1. Introduction

The oxidation of lipids and proteins is a major concern for the food industry. Oxidation promotes rancidity, decreases product shelf life and imparts negative changes in flavour, texture and colour, which adversely affect consumer acceptability [1]. In addition, many end products of the lipid oxidation process are also potentially detrimental to health, contributing to disease pathogenesis by direct effects on cellular and genomic stability or modulating major pathways of cell signalling and gene expression [2,3]. For example, aldehydes, such as malondialdehyde, which are derived primarily from the oxidation of *n*-3 and *n*-6 polyunsaturated fatty acids, are atherogenic and putative mutagens and carcinogens [4,5,6]. Similarly, some advanced lipid oxidation products (ALEs) formed in foods by the reaction of protein with lipid oxidation derived peroxyl and carbonyl compounds may promote inflammation, fibrosis and atypical cell proliferation [7].

Susceptibility of foods to oxidation depends in part on the degree of unsaturation of the fatty acids present. In general, products containing saturated and monounsaturated fats have greater oxidative stability compared with those rich in polyunsaturated fats [8]. In addition to the fatty acid composition, inhibition of oxidation of food lipids is also dependent on the presence of phenolic compounds with antioxidant activity. Such phenolic structures may chelate reactive iron [9] and also confer multiple reductive capacities [10], donating hydrogens or electrons to inhibit the initiation and propagation of lipid oxidation [11]. 

The use of synthetic phenolic antioxidants, such as propyl gallate, butylated hydroxyanisole (BHA) and butylated hydroxytoluene (BHT) by the food industry has declined as consumer preference for natural alternatives has increased [12]. Consequently, there continues to be great interest in identifying and evaluating phenolic antioxidants from plant sources to increase the oxidative stability of food products, such as ground meat patties. Examples include individual compounds or extracts derived from herbs, spices, teas, olives, capers, pine bark, grapes, canola meal and soft fruit [13,14,15,16,17,18,19,20,21]. Less attention has been directed to the use of dried vegetable powders normally used to impart colour and flavour to food products. Such powders are likely to be rich sources of natural compounds with antioxidant activity. Moreover, their consumption in cooked products also may impart additional health benefits, as epidemiological studies strongly implicate inadequate vegetable consumption as a risk factor for heart disease and several cancers [22,23].

The aim of the present study was to assess whether the incorporation of dried powders of widely available vegetables (beetroot, broccoli, carrot, celery, green pea, onion, red pepper, spinach, swede, tomato and yellow pea) contributed to the potential health benefit of turkey meat patties following cooking by improving oxidative stability and decreasing the formation to potentially toxic products of protein and lipid oxidation. 

## 2. Experimental Section

### 2.1. Preparation of Turkey Patties

The basic patty consisted of 10 g of vitamin E stripped corn oil (MP Biochemicals, Illkirch, France) and 145 g of lean, raw turkey meat. Commercially available (JL Priestly & Co Ltd., Lincolnshire, UK) dried powders (beetroot, broccoli, carrot, celery, green pea, onion, red pepper, spinach, swede, tomato and yellow pea) were added (10 g) to the basic burger. The ingredients were thoroughly mixed, formed in to a round, flat patty, placed on a cling-filmed board and chilled. The patties were then individually cooked by heating 5 g of the corn oil in a small frying pan, then adding the burger and frying for 10 min with turning until thoroughly cooked. The patties were then allowed to cool and kept at −20 °C until freeze dried. They were then vacuum packed and stored at −40 °C. When required for analysis, burgers were freezer-milled to a fine, uniform powder. 

### 2.2. Compositional Analysis of Turkey Patties

Routine analytical procedures [24] were employed to determine total fat, protein (as nitrogen), fibre (non-starch polysaccharides), total carbohydrate and energy content of the patties. Fatty acid methyl esters were determined by gas liquid chromatography [25]. Several micronutrients with recognized antioxidant activity were also quantified: vitamin E (α- and γ-tocopherols), carotenoids (α-carotene, β-carotene, β-cryptoxanthin, lutein, lycopene) and vitamin C were analysed by high performance liquid chromatography (HPLC) [26,27]. Measurement of “free” and total phenols in the patties was based on the method described by Vinson *et al*. [28], values being quantified as gallic acid equivalents. The phenolic acids were extracted as described previously [29] and their concentrations determined by liquid chromatography mass spectrometry (LC-MS) using as Agilent 1100 HPLC system (Agilent Technologies, Wokingham, UK) system coupled to a an ABI 3200 triple quadrapole instrument fitted with a Turbo-Ion Spray source (Applied Biosystems; Warrington, UK) using previously described procedures.

### 2.3. Determination of Oxidative Stability of Patties

The effects of the vegetable powders on the oxidative stability of the patties was determined with a 743 Rancimat (Metrohm, Herisau, Switzerland). In brief, this accelerated oxidation method determines the change in conductivity arising from the presence of the oxidation products generated in a heated and aerated sample. An induction time is then interpolated; the longer the induction time, the greater the oxidative stability of the sample. Samples of freezer milled dried patties (3 g) were added to reaction tubes, and oxidative stability was determined following optimization of the manufacturer’s instructions [30] (temperature 50 °C; gas flow rate 7 L/min). The determination of protein carbonyls in the patties [31] followed incubation (25 °C; 10 min) of the samples (0.1 g) with KCl (0.15 M; 1 mL) containing ferrous sulphate (1 mM) and H_2_O_2_ (1 mM) [18]. The total antioxidant capacity of supernatants of the milled patties (1 g added to 24 mL isotonic saline, centrifuged 3500 × *g*, 1 h, 4 °C) was measured using the HORAC assay kit (Oxford Biomedical Research, Oxford, UK), which is based on delayed hydroxyl radical-mediated quenching of fluorescein by compounds in the sample with antioxidant activity [32].

### 2.4. Statistical Analysis

Proximate analyses were conducted on duplicate samples. All other determinations were performed in triplicate and are presented as the mean ± SEM. The statistical significance of differences between the plain burger and burgers with the vegetable powders individually was assessed by Students’ *t*-test. Correlations between various parameters were determined from simple regression analysis to assess potential relationships between variables. 

## 3. Results

### 3.1. Composition of Patties

Patties weighed approximately 150 g, and the dry matter content was 60%. Fat and protein were the dominant macronutrients in the patty, the plain version containing approximately 30% and 67%, respectively. The addition of the powders generally increased carbohydrate by up to 6% (e.g., yellow pea) and fibre by up to 4% (e.g., broccoli) (Table 1). Fat content was unaffected, reflecting the trace amounts present in the incorporated vegetable powders (data not shown). The main fatty acids in the patties were palmitic (C16:0, ≈13.5%), oleic (C18:1(*n*-9), ≈30%) and linoleic acid (C18:2(*n*-6), ≈48%). 

**Table 1:** Macronutrient composition of the patties. Proximate analysis values are the means of duplicate determinations on a dry weight basis. Percentage dry matter = 60%; protein = nitrogen × 6.25; CHO, carbohydrate, calculated “by difference”. Fibre was determined as non-starch polysaccharides (NSP); gross energy (GE) was obtained by bomb calorimetry.

Patty Type	Cooked weight (g)	Fat (g 100 g^−1^)	Protein (g 100 g^−1^)	CHO (g 100 g^−1^)	Fibre (g 100 g^−1^)	GE (kJ 100 g^−1^)
Plain	142	28.3	67.3	0.62	0.53	2543
Carrot	149	23.6	62.6	4.78	2.59	2363
Swede	146	25.6	59.7	4.07	3.67	2443
Broccoli	150	24.7	63.7	1.20	4.08	2305
Celery	148	24.8	61.8	3.78	3.63	2423
Beetroot	148	24.1	63.3	4.89	2.43	2327
Spinach	150	24.6	64.9	0.53	2.68	2437
Yellow pea	151	24.7	64.6	6.18	2.46	2412
Onion	149	25.4	60.5	2.90	2.22	2527
Red pepper	147	25.2	61.3	2.05	2.32	2532
Green pea	148	24.6	62.5	5.69	2.29	2279
Tomato	145	21.9	65.5	2.69	1.44	2264

Unsurprisingly, incorporation of the vegetable powders markedly increased the content of phytochemicals and non-nutritive phytochemicals. Tocopherols, vitamin C and carotenoids markedly increased compared with the plain patties. For example, addition of tomato and red pepper powders increased vitamin C and α-tocopherol contents by 10- and 4-fold, respectively (Table 2); the tomato-based patty was particularly rich in lycopene, and the addition of spinach markedly increased lutein levels (Table 3).

**Table 2:** Selected compounds in patties with potential antioxidant activity and total antioxidant capacity. Values are the mean ± SE of three determinations. GA is the gallic acid equivalents.

Patty Type	Total Phenols (mg GA 100 g^−1^)	Free Phenols (mg GA 100 g^−1^)	Vitamin C (µg 100 g^−1^)	α-Tocopherol (µg 100 g^−1^)	γ-Tocopherol (µg 100 g^−1^)	HORAC Value (µM GA mL^−1^)
Plain	629 ± 10	225 ± 34	305 ± 18	97 ± 6	86 ± 2	11.2 ± 0.7
Carrot	1065 ± 31	300 ± 12	3712 ± 52	184 ± 16	99 ± 1	9.3 ± 0.5
Swede	883 ± 15	256 ± 39	2901 ± 142	114 ± 10	110 ± 2	9.0 ± 0.2
Broccoli	806 ± 22	251 ± 15	19,673 ± 560	708 ± 8	108 ± 4	11.5 ± 0.7
Celery	685 ± 36	258 ± 12	6742 ± 374	166 ± 8	92 ± 1	9.6 ± 0.6
Beetroot	940 ± 20	295 ± 12	6913 ± 472	136 ± 2	93 ± 4	9.7 ± 0.2
Spinach	1137 ± 30	347 ± 8	613 ± 31	838 ± 65	212 ± 23	13.9 ± 1.2
Yellow pea	917 ± 8	232 ± 8	2637 ± 28	157 ± 5	238 ± 8	10.6 ± 0.7
Onion	1067 ± 32	246 ± 19	4780 ± 299	147 ± 4	105 ± 1	8.7 ± 1.4
Red pepper	1159 ± 22	348 ± 5	14,436 ± 807	4503 ± 382	403 ± 8	14.7 ± 1.23
Green pea	737 ± 14	229 ± 4	12,464 ± 484	80 ± 12	185 ± 1	9.6 ± 0.1
Tomato	1232 ± 33	305 ± 4	39,091 ± 538	1794 ± 200	231 ± 18	13.9 ± 1.23

**Table 3:** Concentration of individual carotenoids in patties. Values are the mean ± SE of three determinations. Lutein determination includes zeaxanthin isomer. ND, not detected.

Patty Type	Lutein (µg 100 g^−1^)	β-cryptoxanthin (µg 100 g^−1^)	Lycopene (µg 100 g^−1^)	α-carotene (µg 100 g^−1^)	β-carotene (µg 100 g^−1^)
Plain	0.5 ± 0.1	ND	ND	ND	ND
Carrot	1.8 ± 0.2	1.0 ± 0.1	2.0 ± 0.1	13 ± 4	22 ± 1
Swede	5.0 ± 0.1	ND	4.0 ± 0.3	ND	1.0 ± 0.1
Broccoli	87 ± 5	ND	3.6 ± 0.2	2 ± 0.3	58 ± 4
Celery	0.7 ± 0.05	ND	ND	ND	ND
Beetroot	1.1 ± 0.1	ND	ND	2 ± 0.2	1.0 ± 0.1
Spinach	499 ± 36	ND	12 ± 0.8	1.8 ± 0.1	236 ± 7
Yellow pea	2.0 ± 0.1	ND	ND	ND	ND
Onion	0.9 ± 0.2	ND	ND	ND	ND
Red pepper	33 ± 0.2	26 ± 8	ND	4.5 ± 0.4	115 ± 3
Green pea	18 ± 0.1	ND	10 ± 0.2	ND	8.6 ± 0.1
Tomato	39 ± 2	ND	1842 ± 48	1.7 ± 0.1	206 ± 2

**Figure 1:**
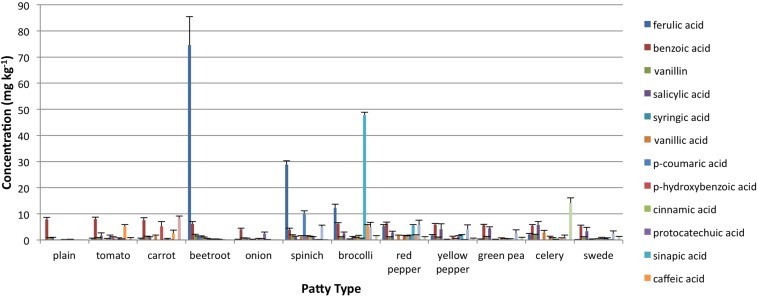
Phenolic compounds detected in the patties by liquid chromatography mass spectrometry.

MS analysis also indicated the presence of several simple phenolic compounds in the patties (Figure 1). Benzoic acid was present in similar concentrations (≈0.05 mol/kg) in all patty types, whereas those with beetroot, spinach and broccoli were the only ones where concentrations of individual phenolics exceeded 30 mg/kg (p-coumaric, ferulic and syringic acid). The presence of compounds with antioxidant potential was reflected in the measurements of total antioxidant capacity (Table 2). HORAC values of the patties were significantly correlated with values for free phenolics (*r* = 0.685; *p* < 0.02), α-tocopherol (*r* = 0.793; *p* < 0.01) and γ-tocopherol (*r* = 0.756; *p* < 0.01).

### 3.2. Oxidative Stability

**Figure 2:**
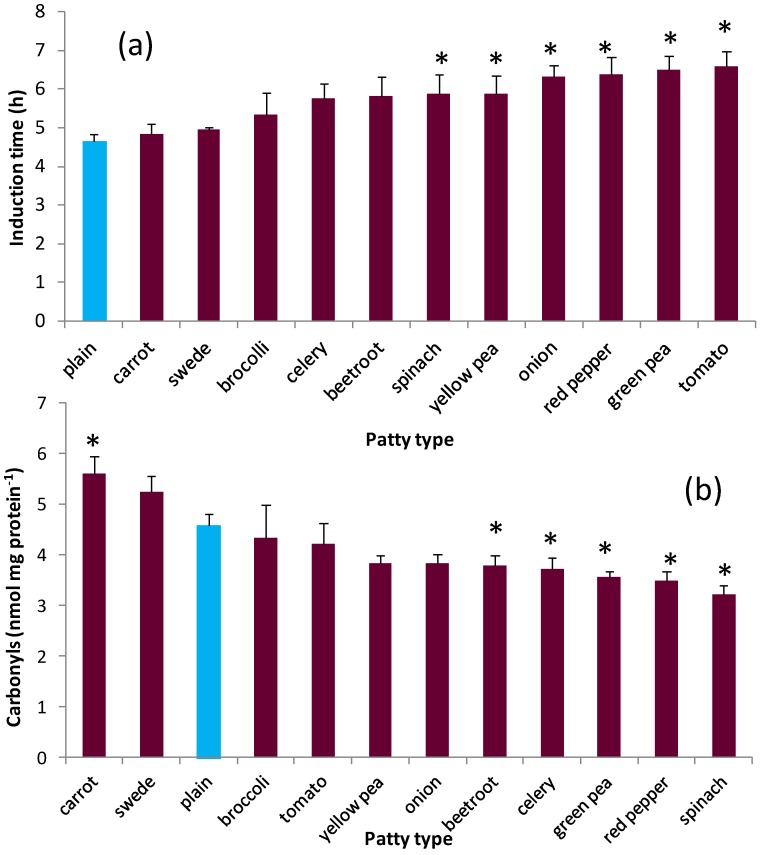
Oxidative stability of patties assessed as (**a**) induction time and (**b**) protein carbonyl content. * Denotes significantly different (*p* < 0.05) from a plain patty containing no freeze dried vegetable powder. Values are the means ± SE (*n* = 3).

In the accelerated oxidation system, the evaluation algorithm of the oxidation curve (induction time) of the basic patty was 4.67 ± 0.16 h. Six of eleven freeze dried powders significantly (*p* < 0.05) improved oxidative stability (spinach < yellow pea < onion < red pepper < green pea < tomato) by 20%–30% (Figure 2a). Inclusion of some the vegetable powders also significantly decreased (*p* < 0.05) the presence of oxidized proteins (determined as carbonyls) when the freeze dried patties were incubated with ferrous sulphate and H_2_O_2_ (beetroot < celery < green pea < red pepper < spinach). There was a significant statistical relationship between induction times and carbonyl contents (*r* = −747; *p* < 0.01), although the inclusion of powdered carrot appeared to have a significant |pro-oxidant effect with respect to carbonyl content (Figure 2b). No multiple testings were done to the results in Figure 2, as there was a clear pattern of significant differences exceeding the 5% Type 1 error rate. However, if such adjustments were done by Tukey or Bonferroni, one significant difference in induction time (yellow pea) would be lost. Regression relationships between the measures of oxidative stability and individual compounds (α-tocopherol, γ-tocopherol, vitamin C) or total phenols did not attain statistical significance.

## 4. Discussion

Increased consumption of plant-based foods is generally recognized as decreasing morbidity from diet-related diseases, such as cardiovascular disease, diabetes and several cancers [22,23]. The mechanisms for these protective effects remain unclear, but are ascribed in part to compounds with antioxidant-related activities, such as tocopherols, vitamin C, carotenoids and phenolics [33]. Despite numerous health promoting campaigns, consumption of fruit and vegetables remains sub-optimum, 78% of populations in low and middle-income countries consuming less than the minimum recommended five daily servings [34]. Drying procedures (e.g., freeze drying) retain many of the nutrients found in the original primary product [35]. Consequently, inclusion of dried vegetable powders within reformulated processed meat products, such as patties, increases their content of natural antioxidants (Figure 1 and Table 2). Such increased antioxidant capacity (Table 2) may contribute to the prevention of diet related diseases. 

In addition, vegetable powders are likely to contain thousands of bioactive compounds with potential health benefits that do not necessary invoke an antioxidant function. *In vitro* studies indicate that many phytochemicals, such as sterols, phenolics, nitrogen containing alkaloids and sulphur compounds, have diverse activities in mammalian systems [36]. These include the inhibition of carcinogen activation, induction of hepatic detoxification pathways, enhancement of immune responses, induction of apoptosis, modification of lipid profiles, anti-inflammatory effects and nitric oxide-mediated vasodilation [37]. For example, some of the phenolics identified in the patties are reported to have marked anti-inflammatory effects [38], and carotenoids and tocopherols are involved in signalling cascades, gene expression and membrane processes [39,40].

Inclusion of vegetable powders in the formulation of processed meat products may have the additional benefit of inhibiting the formation of potentially deleterious products of lipid and protein oxidation during cooking. Such oxidative products may be a risk to human health [7,41] as, in excess, they may exert diabetogenic and nephrotoxic effects, induce low-grade inflammation and promote atherogenesis [42]. In the present study, several vegetable powders significantly improved oxidative stability as estimated by the Rancimat method of assessing the formation of mainly volatile carboxylic acids and the Fe III-stimulated production of protein carbonyls. Such effects could not be definitively ascribed to tocopherols, ascorbate or total phenols in the patties, despite their known reductive capacities in chemical systems [43]. Consequently, other undetermined structures in the powders, such as coumarins and tannins, may more markedly contribute to the oxidative stability of the patties. An exception was carrot powder, which significantly increased the protein carbonyl content. The reason for this apparent effect is unclear, but may reflect the presence of unidentified pro-oxidant compounds or the formation of reactive species through the interaction of carrot specific sugars with carbonyl groups resulting from complex Maillard reaction chemistry that may arise when the patty is cooked [44]. Use of other determinants of oxidation, such as thiobarbituric acid reactive substances (TBARS), may elucidate this further. In addition, other factors that interfere in the physic-chemical characteristics of the product (e.g., water-lipid interfaces, lipophilic and hydrophilic antioxidants differing in behaviour in the food matrix and concentration effects promoting pro-antioxidant effects of antioxidant compounds) may also play a role.

Several synthetic phenolics, such as BHT (E321), BHA (E320) and propyl gallate (E310), are used by the food industry to delay oxidation of products. However, consumer antipathy towards synthetic additives to foods and adverse toxicological reports have led to the identification and characterization of numerous potential replacements from botanical sources [45]. A natural origin does not, of course, imply suitability for human consumption, and few natural compounds and extracts with antioxidant properties have as yet been accepted as “generally recognized as safe (GRAS)” substances by regulatory bodies for use in food products [46]. Use of dried vegetable powders in processed foods not only obviates safety concerns, but may allow manufacturers to decrease the amount of synthetic antioxidants in their products. It may also create additional or new uses for vegetable produce not selected for direct sale, due to poor shape, colour or size and, hence, contribute to reducing the one third of food produced annually that is lost and wasted globally [47]. However, quality control may be an issue, as the relative contribution of the many antioxidant phytochemicals to inhibiting oxidation of the patties will not only depend on the species of vegetable from which the powder is derived, but also on factors, such as variety, growing conditions, degree of maturation and storage conditions. 

The contribution of individual patties containing dried powders may be insufficient to markedly contribute to the recommended dietary intakes of micronutrients associated with health benefits of plant-based foods. Further studies may elucidate whether widespread incorporation of dried vegetable powders by the meat processing industry could have disease preventative effects by raising the intakes of plant-based material in populations, particularly those who have habitually low fruit and vegetable intakes.

## 5. Conclusions

Reformulation of processed meat products to include dried vegetable powders offers an alternative strategy to increase intake of plant-based foods, as the effectiveness of policy-driven initiatives aimed at altering dietary habits and food purchase patterns of the consumer is behaviourally problematical [48,49]. Such an approach may not only decrease intake of potentially toxic meat-derived oxidation products, but may also confer additive and synergistic benefits from the complex mixture of phytochemicals present that may not be gained when a purified phytochemical or extract is used to replace a synthetic antioxidant. However, more studies are needed, such as long-term freezing storage studies, before making a recommendation to include dried vegetable powders to processed meat products. Important issues, such as sensory characteristics of the products and consumer acceptability, also need to be addressed.
